# Epidemiology and infection control of vancomycin-resistant enterococci at a German university hospital: A three-year retrospective cohort study

**DOI:** 10.1371/journal.pone.0297866

**Published:** 2024-02-26

**Authors:** Adrian Jochim-Vukosavic, Frank Schwab, Leonard Knegendorf, Dirk Schlüter, Franz-Christoph Bange, Ella Ebadi, Claas Baier

**Affiliations:** 1 Hannover Medical School, Institute for Medical Microbiology and Hospital Epidemiology, Hannover, Germany; 2 Institute of Hygiene and Environmental Medicine, Charité—University Medicine, Berlin, Germany; University of Tripoli, LIBYA

## Abstract

Vancomycin-resistant enterococci (VRE) occur in hospitalized patients, causing both infection and colonization. In recent years, there has been an increase in VRE in German and other hospitals, raising the question of how to control this epidemic best. To better understand the specific epidemiology and to guide infection control, we conducted a retrospective cohort study analyzing all patients with VRE at Hannover Medical School, a tertiary university clinic in Germany that specializes in solid organ transplantation. Epidemiologic and clinical characteristics of patients with VRE from 2015–2017 were collected. Basic epidemiologic parameters, including VRE incidence and incidence density, were calculated. Independent risk factors for nosocomial VRE infection compared to colonization were assessed using a logistic regression model. There were 1,492 VRE cases corresponding to 822 individual patients. The incidence was 0.8 VRE cases per 100 cases. A total of 536 (35.9%) of the 1,492 VRE cases were acquired nosocomially. Of the 1,492 cases, 912 cases had VRE-positive samples (894 *Enterococcus* (*E*.) *faecium* and 18 *E*. *faecalis*) in our hospital laboratory and the remaining cases were known VRE carriers. The *vanB*-phenotype was observed in 369 of the 894 (41.3%) *E*. *faecium* isolates and in 6 of the 18 (33.3%) *E*. *faecalis* isolates. There was an increase over time in the *vanB*-phenotype proportion in *E*. *faecium* (2015: 63 of 171, 36.8%, 2016: 115 of 322, 35.7% and 2017: 191 of 401, 47.6%). A total of 107 cases had a VRE infection (7.2% of all VRE cases) according to the criteria of the German National Reference Center for Surveillance of Nosocomial Infections. The remaining cases were only colonized. Among other factors, leukocytopenia (<1,000/μL), the use of a central venous catheter and the visceral surgery medical specialty were independently associated with nosocomial VRE infection. VRE imposed a relevant and increasing infection control burden at our hospital. Nosocomial VRE infection was predominantly found in certain medical specialties, such as hematology and oncology and visceral surgery. Infection control efforts should focus on these highly affected patient groups/specialties.

## Introduction

In Germany, a rise in the frequency of healthcare-associated vancomycin-resistant enterococci (VRE) has been observed in recent years [1–3]. VRE cause both colonization and infection, such as bloodstream infection (BSI) [4]. Some patient groups, such as patients with malignancy, are especially affected by VRE [5], and clinical outcomes may worsen in cases of VRE infection [6]. The increase in VRE in Germany was accompanied by a shift from *vanA*-type resistance to the *vanB*-type [3]. Moreover, there are observations showing that certain lineages have become dominant in the German healthcare sector, such as *Enterococcus* (*E*.) *faecium* ST117/CT71 [7–9]. The German National Reference Centre (NRC) for Enterococci recently published a detailed overview of the epidemiologic developments regarding VRE in the recent decades [3]. The spread of VRE is not limited to Germany but rather a global challenge [10]. The World Health Organization has put vancomycin-resistant *E*. *faecium* on the priority list for research and development of new antibiotics for antibiotic-resistant bacteria [11]. VRE are important nosocomial pathogens that can cause hospital outbreaks [12, 13], for instance due to the ability to persist on hospital equipment and surfaces [14]. Moreover, antibiotic therapy may promote VRE acquisition [5], and antibiotic stewardship efforts seem necessary to sustainably address the nosocomial spread of VRE [15].

In summary, an emerging problem for hospitals and for hospital epidemiologists and infection control practitioners in Germany and worldwide is to find appropriate measures to prevent nosocomial VRE acquisition and infection. Several countries have published national guidelines on how to prevent VRE, and these are an important basis for local decision making [16–18]. However, the necessity of some infection control measures, such as isolation, is still a subject of debate [19]. Currently in Germany, hospitals often have more or less different approaches regarding infection control of VRE. Against this background, we attempted to better understand the local epidemiology of VRE by conducting a retrospective cohort study including all patients with VRE from 2015–2017 at Hannover Medical School (Germany), a tertiary university clinic that specializes in solid organ transplantation.

## Methods

### Setting and VRE infection control policy

Hannover Medical School is a university clinic in northern Germany with approximately 1,500 patient beds for the treatment of adult and pediatric patients. During the study period, isolation of VRE patients (single rooms or cohorting) was recommended. Healthcare staff wore personal protective equipment (gowns and gloves) during contact with VRE patients. After discharge, the patient rooms were disinfected by qualified cleaning staff using commercial bactericidal agents. There was no hospital-wide systematic (admission or prevalence) active VRE screening in place during the study period. If a screening took place (e.g., prior to hematopoietic stem cell transplantation), rectal swabs were usually taken. Antiseptic bathing (e.g., with chlorhexidine or octenidine-dihydrochloride) was not recommended for VRE patients during the study period in our institution.

### Study design and definitions

A retrospective cohort study was conducted including all inpatients with VRE (colonization and/or infection; species: *E*. *faecium* and *faecalis*; January 2015 to December 2017, i.e., over 36 months). VRE patients and the corresponding hospital stays (VRE cases) were identified using inhouse infection control software and the laboratory information system. A VRE patient/case either had a VRE-positive sample during the hospital stay and/or was labeled as a known VRE carrier in the infection control software (due to VRE-positive samples in previous hospital stays or in other hospitals). Clinical and demographic data were collected from patient charts. The data were analyzed from April 2020 to December 2022. During data collection, the data were immediately pseudonymized. VRE infections were classified using criteria provided by the German National Reference Center for Surveillance of Nosocomial Infections [20], which are based on the well-established CDC/NHSN definitions [21]. Briefly, the definition includes the detection of VRE in samples taken for infection diagnostics and, for some types of infection, this is combined with other parameters such as the presence of clinical signs of infection. Patients/cases not matching the infection criteria were classified as colonized. In general, a VRE acquisition (colonization/infection) on day 3 or later of the hospital stay without a known history of VRE was defined as hospital-acquired (nosocomial). The number of patients, patient hospital stays (cases) and patient days (number of days in the hospital) were provided by the controlling department of Hannover Medial School.

The ethics committee of the Hannover Medical School approved this study (No. 8987_BO_K_2020). Given that this was a retrospective, quality-assuring study, the need for informed consent was waived by the Ethics Committee and the Data Protection Commissioner of Hannover Medical School.

### Statistical analysis

The incidence and incidence density of VRE were calculated as the number of VRE cases per 100 cases and per 1,000 patient days, respectively. Epidemiological and clinical characteristics of cases with nosocomial VRE infection and VRE colonization were compared. Differences between the two groups were tested using the chi-square test for categorical parameters and the Wilcoxon rank sum test for continuous parameters. Numbers and percentages (categorical parameters) as well as medians, means and interquartile ranges (continuous parameters) were calculated.

To determine independent factors (parameters) that are associated with developing a nosocomial VRE infection (compared to being/remaining VRE-colonized only), a multivariate analysis was carried out using a logistic regression model by stepwise forward variable selection. The significance level for the logistic regression was 0.05. That means, parameters with a p value <0.05 remain in the model and parameters with p value ≥0.05 were removed. For the logistic regression model, parameters that occurred during the “time at risk” were included. For being/remaining VRE-colonized, the “time at risk” was defined as either i) days from the timepoint of the first VRE colonization sample to discharge or ii) days from admission to discharge (for known VRE carriers). For nosocomial VRE infection, the “time at risk” was defined as either i) days from admission to the onset of nosocomial VRE infection (for those cases who directly developed a nosocomial VRE infection without being colonized in advance) or ii) days from the timepoint of the first VRE colonization sample to the onset of nosocomial VRE infection (for those cases who were VRE-colonized in advance of nosocomial VRE infection). To count the days, the day of admission/discharge (counted as a full day) and the day of collection of the positive VRE specimen (colonization and/or infection) were used as needed according to the above definition.

All statistical test results were considered significant at p < 0.05. The analyses were performed with SPSS 26 (IBM SPSS statistics, Somer, NY, USA) and SAS 9.4 (SAS Institute, Cary, NC, USA).

## Results

### Epidemiology

**Fig 1:**
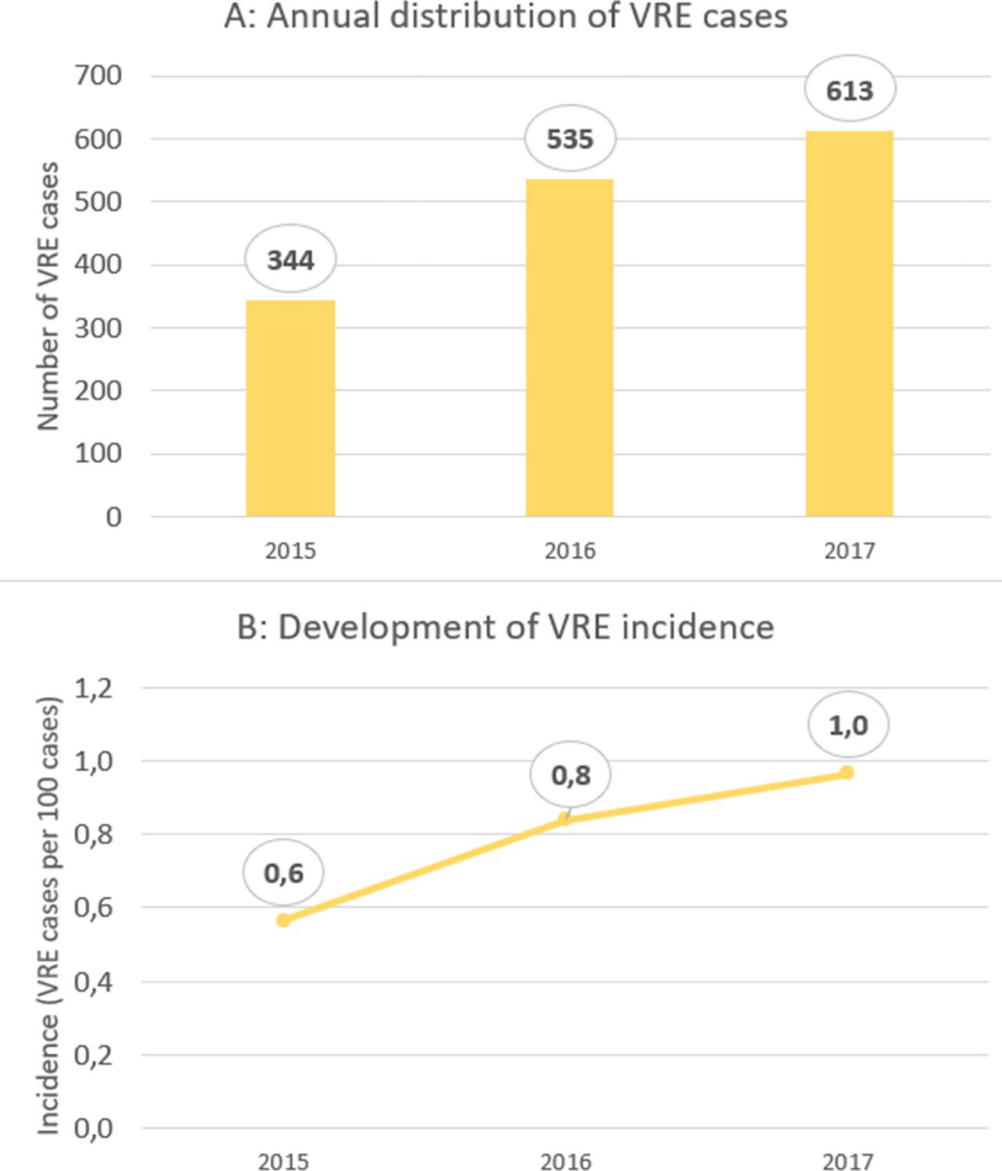
A: Annual distribution of VRE cases. B: Development of VRE incidence.

In the study period, 188,332 cases (corresponding to 137,314 individual patients; i.e., an average of 1.4 cases per patient) were recorded. These cases generated 1,381,806 patient days (mean length of stay: 7.3 days per case). There were 1,492 VRE cases corresponding to 822 individual VRE patients (1.8 cases per VRE patient). This resulted in an incidence of 0.8 VRE cases per 100 cases and an incidence density of 1.1 VRE cases per 1,000 patient days. Regarding individual patients, VRE was detected in 0.6% of all patients. The mean length of stay in the VRE cases was 26.3 days. Fig 1 illustrates the annual distribution of the VRE cases showing a continuous increase in total numbers and incidence in the study period.

A total of 536 (35.9%) of the 1,492 VRE cases were acquired nosocomially and 687 (46.0%) were female. The mean age of the VRE cohort was 55.4 years, and 63 cases (4.2%) were aged 18 and below. A total of 1,452 (97.3%) VRE cases were attributable to *E*. *faecium*, 38 (2.5%) to *E*. *faecalis* and two cases (<0.5%) to both species.

### Microbiologic characteristics

In 912 of the 1,492 VRE cases (61.1%), there were VRE-positive samples in our hospital’s laboratory (the remaining 580 cases were known VRE carriers). The two most common positive sample sites (sample site copy strains eliminated) were rectal/stool (572 of 912, 62.7%) and urine (310 of 912, 34.0%). 894 samples were *E*. *faecium*, and 18 samples were *E*. *faecalis*. The *vanB*-phenotype (in vitro resistance to vancomycin and in vitro susceptibility to teicoplanin) was observed in 369 of the 894 (41.3%) *E*. *faecium* isolates and in 6 of the 18 (33.3%) *E*. *faecalis* isolates. There was an increase over time in the *vanB*-phenotype proportion in *E*. *faecium* (2015: 63 of 171, 36.8%; 2016: 115 of 322, 35.7% and 2017: 191 of 401, 47.6%). Nine (1.0%) of the 912 isolates were linezolid resistant (all *E*. *faecium*). Phenotypic linezolid resistance was increasing over time (2015: 1 of 171, 0.6%; 2016: 3 of 322, 0.9% and 2017: 5 of 401, 1.2%).

### VRE infection and colonization

There were 107 cases with VRE infection (7.2% of all VRE cases), of which 84 (78.5% of all VRE infections) were acquired in the hospital (nosocomial). A total of 102 (95.3%) VRE infections were caused by *E*. *faecium*. The 84 nosocomial VRE infection cases consisted of 36 bloodstream infections, 31 postoperative/soft tissue infections, 12 urinary tract infections, 3 intraabdominal infections, 1 endocarditis and 1 ventriculitis. Fifteen (17.9%) of the 84 nosocomial VRE infection cases were colonized with VRE prior to infection onset. The median length from the first VRE colonization sample to infection was 15 days in these cases. These 15 VRE infection cases with prior VRE colonization included 9 bloodstream infections, 3 postoperative/soft tissue infections and 3 urinary tract infections. Differentiated by infection type, the median length from the first VRE colonization sample to infection was 16 days for bloodstream infections, 15 days for postoperative/soft tissue infections, and 5 days for urinary tract infections.

**Fig 2:**
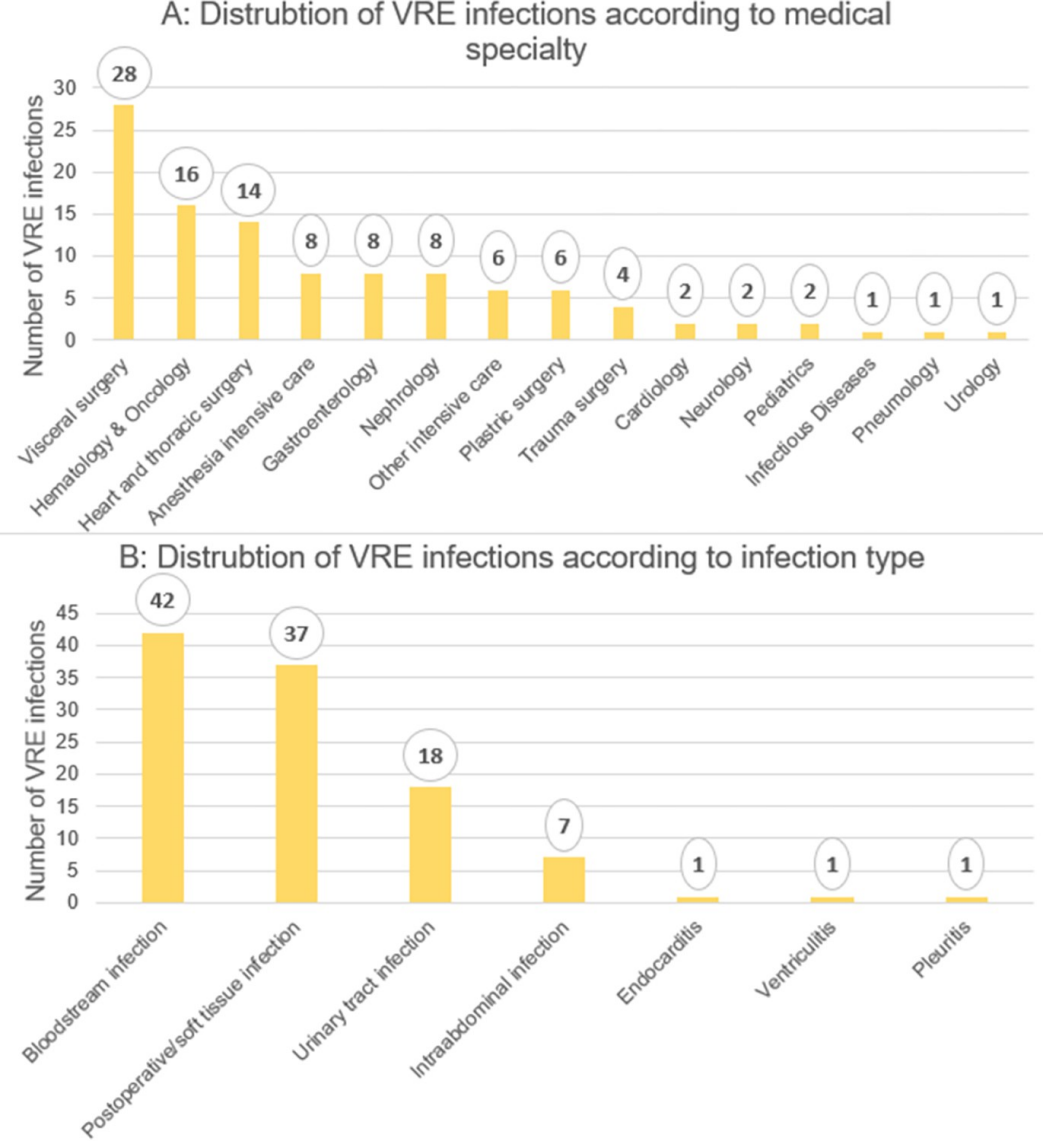
A: Distribution of VRE infections according to medical specialty. B: Distribution of VRE infections according to infection type. Overall, 107 VRE infections occurred.

Fig 2 shows the distribution of all VRE infection cases according to infection type and clinical specialty. Infections occurred most frequently in visceral surgery, hematology and oncology and heart and thoracic surgery. The three most common types of infections were bloodstream infections, postoperative/soft tissue infections and urinary tract infections. 1,385 VRE cases (92.8%) were colonized only.

### Comparison of nosocomial VRE infection and colonization

The 84 cases with nosocomial VRE infection were compared to 1,385 VRE cases with colonization only. Cases with nosocomial VRE infection had a significantly longer median length of stay (43 days vs. 15 days, p<0.001), and the proportion of deaths was also significantly higher in the infection group (31% vs. 9.5%, p<0.001). For example, the medical specialty visceral surgery was significantly more represented in the nosocomial infection cohort than in the colonization cohort (28.6% vs. 12.1%, p<0.001). Among other factors, leukocytopenia (20.2% vs. 10.3%, p = 0.005), a hemoglobin level < 8g/dL (60.7% vs. 33.4%, p<0.001), the use of a central venous catheter (89.3% vs. 52.3%, p<0.001), the use of a drainage (48.8% vs. 29.5%, p<0.001) and a urinary catheter (69.0% vs. 44.0%, p<0.001) and the parameters invasive ventilation (44.0% vs. 16.8%, p<0.001) as well as any surgery (63.1% vs. 32.3%, p<0.001) were also significantly more often found in the nosocomial VRE infection group. All clinical and epidemiologic characteristics of both groups are summarized in Table 1.

**Table 1:** Comparison of epidemiologic and clinical characteristics of cases with nosocomial VRE infection and VRE colonization.

Parameter	Cases with VRE colonization only (n = 1,385; 100%)	Cases with nosocomial VRE infection (n = 84; 100%)	p value*
**Basic epidemiologic information**			
Median length of stay in days [IQR]	15 [7–31]	43 [25–64]	**<0.001**
Median length of “time at risk” in days [IQR]	10 [5–21]	17 [8–64]	**<0.001**
Discharge: Death	132 (9.5%)	26 (31%)	**<0.001**
Discharge: Home	1029 (74.3%)	38 (45.2%)	**<0.001**
Discharge: Transfer to another hospital	224 (16.2%)	20 (23.8%)	0.068
Median number of prior hospital stays [IQR]	4 [1–11]	1 [0–4]	**<0.001**
Median age in years [IQR]	58 [45–68]	59 [46–71]	0.713
Female	639 (46.1%)	37 (44.0%)	0.709
**Distribution of cases according to medical specialty**			
Anesthesia intensive care	32 (2.3%)	6 (7.1%)	**0.007**
Ophthalmology	2 (0.1%)	0 (0%)	0.727
Dermatology	8 (0.6%)	0 (0%)	0.485
Gastroenterology	291 (21.0%)	4 (4.8%)	**<0.001**
Gynecology	4 (0.3%)	0 (0%)	0.622
Hematology & Oncology	246 (17.8%)	13 (15.5%)	0.594
Otorhinolaryngology	8 (0.6%)	0 (0%)	0.485
Heart and thoracic surgery	160 (11.6%)	13 (15.5%)	0.279
Infectious Diseases	15 (1.1%)	1 (1.2%)	0.927
Immunology	1 (0.1%)	0 (0%)	0.805
Other intensive care	63 (4.5%)	5 (6.0%)	0.552
Cardiology	18 (1.3%)	2 (2.4%)	0.406
Oral and maxillofacial surgery	8 (0.6%)	0 (0%)	0.485
Neurology	46 (3.3%)	1 (1.2%)	0.281
Nephrology	160 (11.6%)	3 (3.6%)	**0.024**
Pediatrics	25 (1.8%)	2 (2.4%)	0.703
Plastic surgery	21 (1.5%)	5 (6%)	**0.003**
Pneumology	28 (2%)	0 (0%)	0.188
Psychiatry	1 (0.1%)	0 (0%)	0.805
Trauma surgery	35 (2.5%)	4 (4.8%)	0.216
Urology	44 (3.2%)	1 (1.2%)	0.305
Visceral surgery	168 (12.1%)	24 (28.6%)	**<0.001**
Dentistry	1 (0.1%)	0 (0%)	0.805
**Carriage of multidrug-resistant bacteria prior to VRE acquisition**			
Methicillin-resistant *Staphylococcus aureus*	64 (4.6%)	0 (0%)	**0.044**
Gram-negative bacteria resistant to ciprofloxacin, third generation cephalosporins and piperacillin	211 (15.2%)	5 (6.0%)	**0.020**
Gram-negative bacteria resistant to carbapenems	39 (2.8%)	3 (3.6%)	0.687
**Underlying diseases**			
Heart disease	321 (23.2%)	25 (29.8%)	0.167
Lung disease	375 (27.1%)	30 (35.7%)	0.085
Liver disease	341 (24.6%)	18 (21.4%)	0.509
Gastroenterological disease	581 (41.9%)	44 (52.4%)	0.060
Oncological disease	709 (51.2%)	46 (54.8%)	0.525
Solid neoplasia	167 (12.1%)	14 (16.7%)	0.212
Dermatological disease	133 (9.6%)	14 (16.7%)	**0.036**
Ocular disease	32 (2.3%)	1 (1.2%)	0.501
Vessel disease	422 (30.5%)	22 (26.2%)	0.407
Kidney disease	509 (36.8%)	29 (34.5%)	0.681
Neurological disease	158 (11.4%)	12 (14.3%)	0.423
Gynecological disease	23 (1.7%)	2 (2.4%)	0.620
Urologic disease	267 (19.3%)	21 (25.0%)	0.200
Orthopedic disease	86 (6.2%)	9 (10.7%)	0.103
Thyroid disease	20 (1.4%)	2 (2.4%)	0.492
Amputation	6 (0.4%)	3 (3.6%)	**<0.001**
Ear-Nose-Throat Disease	21 (1.5%)	1 (1.2%)	0.811
Dental disease	20 (1.4%)	3 (3.6%)	0.127
Diabetes mellitus	125 (9.0%)	5 (6.0%)	0.336
Rheumatic disease	18 (1.3%)	3 (3.6%)	0.089
Psychiatric disease	12 (0.9%)	0 (0%)	0.392
**Clinical characteristics**			
Leukocytopenia (<1,000/μL)	143 (10.3%)	17 (20.2%)	**0.005**
Hemoglobin <8g/dL	462 (33.4%)	51 (60.7%)	**<0.001**
Central venous catheter	725 (52.3%)	75 (89.3%)	**<0.001**
Peripheral venous catheter	909 (65.6%)	54 (64.3%)	0.801
Drainage	409 (29.5%)	41 (48.8%)	**<0.001**
Invasive ventilation	233 (16.8%)	37 (44.0%)	**<0.001**
Urinary catheter	610 (44.0%)	58 (69.0%)	**<0.001**
Any surgery	448 (32.3%)	53 (63.1%)	**<0.001**
Solid organ transplantation	21(1.5%)	3 (3.6%)	0.149
Hematopoietic stem cell transplantation	53 (3.8%)	4 (4.8%)	0.667
Chemotherapy	61 (4.4%)	4 (4.8%)	0.877

*2 tailed p value, chi-square test for categorical parameters and Wilcoxon rank sum test for continuous parameters. Significant results are displayed in bold.

VRE = vancomycin-resistant enterococci, ICU = intensive care unit.

### Risk factor analysis for the outcome nosocomial VRE infection

A multivariate analysis was performed to determine independent parameters associated with the development of nosocomial VRE infection (compared to being/remaining VRE colonized only). Leukocytopenia (<1,000/μL), the use of a central venous catheter, a urologic disease, the medical specialties plastic and visceral surgery as well as the parameters any surgery and amputation were independently associated with nosocomial VRE infection. All parameters which were considered in the analysis are listed in the S1 Table. The significant results of the multivariate analysis are summarized in Table 2.

**Table 2:** Significant results of the multivariate analysis comparing nosocomial VRE infection with VRE colonization (outcome: Nosocomial VRE infection; logistic regression analysis).

Parameter	OR (CI95)	p value
Urologic disease	2.325 (1.333–4.057)	0.003
Leukocytopenia (<1,000/μL)	3.321 (1.740–6.338)	<0.001
Central venous catheter	6.187 (2.983–12.833)	<0.001
Specialty plastic surgery	4.919 (1.561–15.496)	0.007
Specialty visceral surgery	2.665 (1.531–4.639)	0.001
Amputation	5.510 (1.078–28.164)	0.040
Any surgery	2.895 (1.716–4.884)	<0.001

OR = odds ratio, CI95 = 95% confidence interval

## Discussion

To inform and optimize the infection control management of VRE, we retrospectively analyzed VRE infection and colonization at a German university clinic over a three-year period. Against the background of a general increase in VRE in Germany [3], we also observed a continuous increase in VRE in our clinic during the study period. The overall occurrence of VRE in our setting (0.6% of all patients) is comparable to results from a study by Simor et al. reporting a median VRE prevalence of 0.5% in Canadian hospitals [22]. Notably, the prevalence of VRE is higher in studies that used active VRE screening approaches. In a study from another German university hospital (2014/15), the VRE colonization prevalence on admission was 1.2% using a cultural screening approach [23]. A Greek study from 2018 found a VRE point prevalence of 13% among inpatients in a tertiary care hospital who were screened with selective culture media [24]. In a study at our own hospital, the VRE prevalence was 23.8% in hematology and oncology for adults using weekly and admission cultural screening in 2018/19 [25].

The burden of VRE in our study was clearly higher than the burden of carbapenem-resistant *Acinetobacter baumannii* or carbapenem-resistant *Klebsiella pneumoniae* in our hospital (both <0.1%; time period 2015–2019) [26].

Similar to Weber et al. [7], we also observed a shift toward the *vanB*-phenotype in *E*. *faecium* isolates going hand in hand with the overall increase in VRE. An additional linezolid resistance in VRE is of concern, because it further limits therapeutical options in case of infection. In our study, the rate of linezolid resistance in VRE was relatively low and comparable to a study from Southern Germany, in which a rate of 1.4% in 2018 was reported [27].

Of note, two thirds of the VRE cases occurred on day 1 or 2 of the hospital stay or the patients were already known VRE carriers (due to positive VRE samples in prior hospital stays). In this context, Boeing et al. published a study describing a risk score (including for instance the items age >60 years and hemato-oncological disease) for the persistence of VRE colonization [28]. This is of particular interest with regard to VRE positive patients who are repetitively hospitalized due to chronic diseases.

Spatial separation (contact precautions) was recommended for VRE patients in our hospital during the study period and thereafter, resulting in significant occupation of isolation capacity (e.g., single rooms). Especially in the winter seasons with respiratory viruses requiring additional isolation capacity, it was and still is challenging to always enable spatial separation of VRE carriers. This circumstance might undermine efforts to prevent nosocomial transmission of VRE in our setting. In fact, one-third of the cases acquired nosocomial VRE in our cohort. It was beyond the scope of this study to assess the most likely VRE acquisition mode for these cases. However, for instance, transmission via VRE patients in shared rooms or the role of prior room occupancy by VRE patients are described in the literature [29, 30]. In another study, Cohen et al. investigated the relationship between nosocomial infections and exposure to roommates and prior bed occupants with the same pathogen [31]. The study included infections caused by VRE and vancomycin-susceptible *E*. *faecium* and *E*. *faecalis* as well as infections caused by other bacterial pathogens (such as *Staphylococcus aureus* and *Acinetobacter baumannii*). The study showed that infected or colonized roommates and prior occupants posed a significant risk for infection and the authors concluded that thorough disinfection measures in particular are needed. In addition, nosocomial VRE acquisition may also occur in the context of hospital outbreaks [32]. High colonization pressure on a ward is also known to be a relevant factor facilitating VRE transmission [33]. Moreover, limited VRE screening sensitivity using rectal swabs [34] and our screening policy in the study period (no general systematic admission screening) might also influence the rate of nosocomial VRE acquisitions. Antibiotic stewardship aspects also need to be considered. Notably, Neumann et al. showed that a combination of whole genome sequencing and contact network modelling is a powerful tool to understand the transmission dynamics of VRE within a hospital [35].

The debate on how to best handle VRE in terms of infection control is ongoing [19]. A report from Switzerland indicates that widespread adoption of comprehensive control measures targeting VRE (including, for instance, isolation) may contribute to the control of VRE in hospitals [17]. A study by Liese et al. showed that patient transfers within a hospital and between health care facilities as well as repeated entries from outside influenced the spread of VRE within an academic tertiary hospital in southwest Germany [36]. The authors therefore emphasize the importance of network strategies. There is also evidence in the literature that the discontinuation of contact precautions for VRE does not lead to an increase in nosocomial VRE infection rates when well-adapted infection control practices are implemented and the baseline infection rate is low [37]. In another report, however, cessation of contact precautions and active screening were associated with an increase in VRE infection rates [38]. In our cohort, approximately 7% of the VRE carriers had a VRE infection, and the majority had a nosocomial onset of infection. The infection rate among the VRE carriers in our cohort is higher than in the Canadian study mentioned above, in which 3.1% of the VRE positive patients had a VRE infection [22]. Others, however, found higher infection rates than we did, for instance, in patients with cancer (13.4% of 179 VRE-positive patients developed a VRE bloodstream infection in a study by Zaas et al.) [39]. In a previous work of ours, we studied VRE infections and colonization in a hematology and oncology ward [25]. In that study, 3% of intestinal VRE carriers had VRE infection. Presumably, the kind of patient cohort observed and the specific setting (e.g., endemic vs. epidemic situation, national VRE burden or the hospital’s VRE screening policy) may influence the actual infection to colonization ratio.

In order to tailor local infection control policies, it is important to analyze the distribution of VRE infections by medical specialty. In our study cohort, many VRE infections were found in visceral surgery, hematology and oncology as well as heart and thoracic surgery. Interestingly, the specialty gastroenterology was strongly represented in the colonization group, but the proportion of infection was rather low. Especially regarding limited isolation and infection control workforce capacities, it seems rational to focus infection control efforts (e.g., teaching, training, audits, isolation) on medical specialties with a high infection burden. Comparing nosocomial VRE infection with VRE colonization, we found in our study some factors that were significantly associated with VRE infection, such as leukocytopenia and the presence of a central venous catheter–both of which are factors often present in hematologic and oncologic patients. Neutropenia and use of a central venous catheter have also been found to be independent risk factors for VRE bloodstream infection in a matched case-control study by Gouliouris et al. [40], who compared patients with and without VRE bloodstream infection. Again, hematologic and oncologic patients must be kept in mind, as they are frequently colonized with VRE, and VRE colonization is often followed by VRE infection in this patient population [5]. The presence of a hemodialysis catheter was reported to be a risk factor for VRE infection among VRE carriers in a study by Kim et al. [41]. Two surgical disciplines (plastic surgery and visceral surgery) and the parameter “any surgery” were significantly associated with nosocomial VRE infection in our cohort. Matching our results regarding visceral surgery, the study by Zaas et al. [39] found gastrointestinal procedures to be an independent risk for VRE infection among VRE-colonized patients. This underlines, that especially in visceral surgery (and in general in all surgical disciplines), consequent infection control efforts and clinical awareness regarding VRE seem warranted. This is of relevance because in our own practical experience, the surgical setting might be underestimated regarding the (increasing) VRE challenge. Of note, solid organ transplantation was not a risk factor for nosocomial VRE infection. The visceral surgery department strengthened VRE infection control measures over time using, for instance, active admission and weekly screening. In summary, the results obtained in this study helped to focus our infection control policy.

Our study has strengths and limitations. Regarding limitations, the current study was conducted in Germany, which may limit its transferability to other settings, for example, due to different structural and human resource conditions. In addition, data collection was retrospective. Moreover, it was beyond the scope of our study to analyze factors associated with VRE-related mortality or to assess the economic impact of VRE. We did not use a comorbidity score to stratify the burden of existing diseases in the VRE colonization and VRE infection groups; however, length of stay and type of hospital discharge may be taken as surrogate parameters. It was not within the scope of this study to determine the molecular characteristics of the VRE isolates. Regarding strengths, we can provide a comprehensive and detailed overview of the VRE burden in a dynamic epidemic situation over a three-year period with approximately 1,500 VRE cases included. Moreover, we provide a detailed insight into the early period of the VRE increase in Northern Germany at a university clinic specialized in solid organ transplantation. Additionally, we analyzed factors independently associated with VRE infection that can be considered by healthcare workers caring for patients with a possible risk of VRE infection.

## Conclusions

VRE imposed a relevant and increasing infection control burden at our tertiary care university hospital. Simultaneously with a common trend found in Germany, we observed an expansion of the *vanB*-phenotype *E*. *faecium* at our center. Nosocomial VRE infection was predominantly found in certain medical specialties, such as hematology and oncology, visceral and heart and thoracic surgery. Bloodstream infections were the most common type of infection. Infection control efforts should focus–especially as human and structural resources for infection control are often limited–on these highly affected patient groups/specialties. Analyzing the specific epidemiology of VRE infection and colonization is essential for guiding these efforts efficiently and effectively.

## Supporting information

S1 Checklist Strobe statement checklist. (DOCX)

S1 Table Parameters considered in the multivariable analysis. (DOCX)

## References

[1] Remschmidt C, Schröder C, Behnke M, Gastmeier P, Geffers C, Kramer TS. Continuous increase of vancomycin resistance in enterococci causing nosocomial infections in Germany—10 years of surveillance. Antimicrob Resist Infect Control. 2018;7:54. 29760912 10.1186/s13756-018-0353-x PMC5937822

[2] Markwart R, Willrich N, Haller S, Noll I, Koppe U, Werner G, et al. The rise in vancomycin-resistant Enterococcus faecium in Germany: data from the German Antimicrobial Resistance Surveillance (ARS). Antimicrob Resist Infect Control. 2019;8:147. doi: 10.1186/s13756-019-0594-3 31485325 PMC6712849

[3] Werner G, Neumann B, Weber RE, Kresken M, Wendt C, Bender JK, et al. Thirty years of VRE in Germany—“expect the unexpected”: The view from the National Reference Centre for Staphylococci and Enterococci. Drug Resist Updat. 2020;53:100732. doi: 10.1016/j.drup.2020.100732 33189998

[4] Brinkwirth S, Martins S, Ayobami O, Feig M, Noll I, Zacher B, et al. Germany’s Burden of Disease of Bloodstream Infections Due to Vancomycin-Resistant Enterococcus faecium between 2015–2020. Microorganisms. 2022;10:2273. doi: 10.3390/microorganisms10112273 36422343 PMC9717732

[5] Alevizakos M, Gaitanidis A, Nasioudis D, Tori K, Flokas ME, Mylonakis E. Colonization With Vancomycin-Resistant Enterococci and Risk for Bloodstream Infection Among Patients With Malignancy: A Systematic Review and Meta-Analysis. Open Forum Infect Dis. 2017;4:ofw246. doi: 10.1093/ofid/ofw246 28480243 PMC5414102

[6] Weber S, Hogardt M, Reinheimer C, Wichelhaus TA, Kempf VAJ, Kessel J, et al. Bloodstream infections with vancomycin-resistant enterococci are associated with a decreased survival in patients with hematological diseases. Ann Hematol. 2019;98:763–73. doi: 10.1007/s00277-019-03607-z 30666433

[7] Weber A, Maechler F, Schwab F, Gastmeier P, Kola A. Increase of vancomycin-resistant Enterococcus faecium strain type ST117 CT71 at Charité—Universitätsmedizin Berlin, 2008 to 2018. Antimicrob Resist Infect Control. 2020;9:109. 32678047 10.1186/s13756-020-00754-1 PMC7364619

[8] Falgenhauer L, Fritzenwanker M, Imirzalioglu C, Steul K, Scherer M, Rhine-Main VREfm study group, et al. Near-ubiquitous presence of a vancomycin-resistant Enterococcus faecium ST117/CT71/vanB -clone in the Rhine-Main metropolitan area of Germany. Antimicrob Resist Infect Control. 2019;8:128. doi: 10.1186/s13756-019-0573-8 31384433 PMC6664515

[9] Falgenhauer L, Preuser I, Imirzalioglu C, Falgenhauer J, Fritzenwanker M, Mack D, et al. Changing epidemiology of vancomycin-resistant Enterococcus faecium: Results of a genome-based study at a regional neurological acute hospital with intensive care and early rehabilitation treatment. Infection Prevention in Practice. 2021;3:100138. doi: 10.1016/j.infpip.2021.100138 34368749 PMC8335922

[10] Shrestha S, Kharel S, Homagain S, Aryal R, Mishra SK. Prevalence of vancomycin-resistant enterococci in Asia—A systematic review and meta-analysis. J Clin Pharm Ther. 2021;46:1226–37. doi: 10.1111/jcpt.13383 33630382

[11] Tacconelli E, Carrara E, Savoldi A, Harbarth S, Mendelson M, Monnet DL, et al. Discovery, research, and development of new antibiotics: the WHO priority list of antibiotic-resistant bacteria and tuberculosis. Lancet Infect Dis. 2018;18:318–27. doi: 10.1016/S1473-3099(17)30753-3 29276051

[12] Gast KB, van Oudheusden AJG, Murk JL, Stohr JJJM, Buiting AG, Verweij JJ. Successful containment of two vancomycin-resistant Enterococcus faecium (VRE) outbreaks in a Dutch teaching hospital using environmental sampling and whole-genome sequencing. Journal of Hospital Infection. 2021;111:132–9. doi: 10.1016/j.jhin.2021.02.007 33582200

[13] Piezzi V, Wassilew N, Atkinson A, D’Incau S, Kaspar T, Seth-Smith HM, et al. Nosocomial outbreak of vancomycin-resistant Enterococcus faecium (VRE) ST796, Switzerland, 2017 to 2020. Eurosurveillance. 2022;27:2200285. doi: 10.2807/1560-7917.ES.2022.27.48.2200285 36695463 PMC9716646

[14] Weber DJ, Anderson D, Rutala WA. The role of the surface environment in healthcare-associated infections. Current Opinion in Infectious Diseases. 2013;26:338–44. doi: 10.1097/QCO.0b013e3283630f04 23743816

[15] Webb BJ, Majers J, Healy R, Jones PB, Butler AM, Snow G, et al. Antimicrobial Stewardship in a Hematological Malignancy Unit: Carbapenem Reduction and Decreased Vancomycin-Resistant Enterococcus Infection. Clin Infect Dis. 2020;71:960–7. doi: 10.1093/cid/ciz900 31751470

[16] Hygienemaßnahmen zur Prävention der Infektion durch Enterokokken mit speziellen Antibiotikaresistenzen. Empfehlung der Kommission für Krankenhaushygiene und Infektionsprävention (KRINKO) beim Robert Koch-Institut. Bundesgesundheitsblatt Gesundheitsforschung Gesundheitsschutz. 2018;61:1310–61. 10.1007/s00103-018-2811-2 30229318

[17] Vuichard-Gysin D, Sommerstein R, Kronenberg A, Buetti N, Eder M, Piezzi V, et al. High adherence to national IPC guidelines as key to sustainable VRE control in Swiss hospitals: a cross-sectional survey. Antimicrob Resist Infect Control. 2022;11:19. doi: 10.1186/s13756-022-01051-9 35090563 PMC8795934

[18] Lepelletier D, Berthelot P, Lucet JC, Fournier S, Jarlier V, Grandbastien B, et al. French recommendations for the prevention of “emerging extensively drug-resistant bacteria” (eXDR) cross-transmission. Journal of Hospital Infection. 2015;90:186–95. doi: 10.1016/j.jhin.2015.04.002 25986165

[19] Vehreschild J, Haverkamp M, Biehl LM, Lemmen S, Fätkenheuer G. Vancomycin-resistant enterococci (VRE): a reason to isolate? Infection. 2019;47:7–11. 30178076 10.1007/s15010-018-1202-9

[20] Nationales Referenzzentrum für Surveillance von nosokomialen Infektionen, Robert Koch-Institut. Definitionen nosokomialer Infektionen für die Surveillance im Krankenhaus-Infektions-Surveillance-System (KISS-Definitionen). 2017. 10.17886/rkipubl-2016-013.2.

[21] Horan TC, Andrus M, Dudeck MA. CDC/NHSN surveillance definition of health care-associated infection and criteria for specific types of infections in the acute care setting. Am J Infect Control. 2008;36:309–32. doi: 10.1016/j.ajic.2008.03.002 18538699

[22] Simor AE, Williams V, McGeer A, Raboud J, Larios O, Weiss K, et al. Prevalence of Colonization and Infection with Methicillin-Resistant *Staphylococcus* *aureus* and Vancomycin-Resistant *Enterococcus* and of *Clostridium difficile* Infection in Canadian Hospitals. Infect Control Hosp Epidemiol. 2013;34:687–93. 10.1086/670998 23739072

[23] Bui MT, Rohde AM, Schwab F, Märtin N, Kipnis M, Boldt A-C, et al. Prevalence and risk factors of colonisation with vancomycin-resistant Enterococci faecium upon admission to Germany’s largest university hospital. GMS Hyg Infect Control. 2021;16:Doc06. doi: 10.3205/dgkh000377 33643773 PMC7894188

[24] Vasilakopoulou A, Karakosta P, Vourli S, Tarpatzi A, Varda P, Kostoula M, et al. Gastrointestinal Carriage of Vancomycin-Resistant Enterococci and Carbapenem-Resistant Gram-Negative Bacteria in an Endemic Setting: Prevalence, Risk Factors, and Outcomes. Front Public Health. 2020;8:55. doi: 10.3389/fpubh.2020.00055 32257988 PMC7093565

[25] Chhatwal P, Ebadi E, Thol F, Koenecke C, Beutel G, Ziesing S, et al. Prospective infection surveillance and systematic screening for vancomycin-resistant enterococci in hematologic and oncologic patients–findings of a German tertiary care center. J Glob Antimicrob Resist. 2020;22:102–5. doi: 10.1016/j.jgar.2020.02.012 32092477

[26] Chhatwal P, Ebadi E, Schwab F, Ziesing S, Vonberg R-P, Simon N, et al. Epidemiology and infection control of carbapenem resistant Acinetobacter baumannii and Klebsiella pneumoniae at a German university hospital: a retrospective study of 5 years (2015–2019). BMC Infect Dis. 2021;21:1196. 34837973 10.1186/s12879-021-06900-3 PMC8627082

[27] Heininger A, Zimmermann S, Bootsveld C, Boutin S, Nurjadi D. Low prevalence of combined linezolid- and vancomycin-resistant Enterococcus faecium from hospital admission screening in an endemic region in Germany. J Glob Antimicrob Resist. 2020;22:646–50. doi: 10.1016/j.jgar.2020.05.003 32439568

[28] Boeing C, Correa-Martinez CL, Schuler F, Mellmann A, Karch A, Kampmeier S. Development and Validation of a Tool for the Prediction of Vancomycin-Resistant Enterococci Colonization Persistence-the PREVENT Score. Microbiol Spectr. 2021;9:e0035621–21. doi: 10.1128/Spectrum.00356-21 34523992 PMC8557884

[29] Zhou Q, Moore C, Eden S, Tong A, McGeer A. Factors Associated With Acquisition of Vancomycin-Resistant Enterococci (VRE) in Roommate Contacts of Patients Colonized or Infected with VRE in a Tertiary Care Hospital. Infect Control Hosp Epidemiol. 2008;29:398–403. doi: 10.1086/587187 18419360

[30] Drees M, Snydman DR, Schmid CH, Barefoot L, Hansjosten K, Vue PM, et al. Prior environmental contamination increases the risk of acquisition of vancomycin-resistant enterococci. Clinical Infectious Diseases. 2008;46:678–85. doi: 10.1086/527394 18230044

[31] Cohen B, Liu J, Cohen AR, Larson E. Association Between Healthcare-Associated Infection and Exposure to Hospital Roommates and Previous Bed Occupants with the Same Organism. Infect Control Hosp Epidemiol. 2018;39:541–6. 10.1017/ice.2018.22 PMC5935247 29486805

[32] Ulrich N, Vonberg R-P, Gastmeier P. Outbreaks caused by vancomycin-resistant Enterococcus faecium in hematology and oncology departments: A systematic review. Heliyon. 2017;3:e00473. doi: 10.1016/j.heliyon.2017.e00473 29322099 PMC5753762

[33] Ford CD, Lopansri BK, Gazdik MA, Webb B, Snow GL, Hoda D, et al. Room contamination, patient colonization pressure, and the risk of vancomycin-resistant Enterococcus colonization on a unit dedicated to the treatment of hematologic malignancies and hematopoietic stem cell transplantation. Am J Infect Control. 2016;44:1110–5. doi: 10.1016/j.ajic.2016.03.044 27287734

[34] D’Agata EMC, Gautam S, Green WK, Tang Y. High Rate of False‐Negative Results of the Rectal Swab Culture Method in Detection of Gastrointestinal Colonization with Vancomycin‐Resistant Enterococci. Clinical Infectious Diseases. 2002;34:167–72. doi: 10.1086/338234 11740703

[35] Neumann B, Bender JK, Maier BF, Wittig A, Fuchs S, Brockmann D, et al. Comprehensive integrated NGS-based surveillance and contact-network modeling unravels transmission dynamics of vancomycin-resistant enterococci in a high-risk population within a tertiary care hospital. PLoS One. 2020;15:e0235160. doi: 10.1371/journal.pone.0235160 32579600 PMC7314025

[36] Liese J, Schüle L, Oberhettinger P, Tschörner L, Nguyen T, Dörfel D, et al. Expansion of Vancomycin-Resistant Enterococcus faecium in an Academic Tertiary Hospital in Southwest Germany: a Large-Scale Whole-Genome-Based Outbreak Investigation. Antimicrob Agents Chemother. 2019;63:e01978–18. doi: 10.1128/AAC.01978-18 30782988 PMC6496047

[37] Martin EM, Colaianne B, Bridge C, Bilderback A, Tanner C, Wagester S, et al. Discontinuing MRSA and VRE contact precautions: Defining hospital characteristics and infection prevention practices predicting safe de-escalation. Infect Control Hosp Epidemiol. 2022;43:1595–602. doi: 10.1017/ice.2021.457 34847970

[38] Johnstone J, Shing E, Saedi A, Adomako K, Li Y, Brown KA, et al. Discontinuing contact precautions for vancomycin-resistant enterococcus (vre) is associated with rising vre bloodstream infection rates in ontario hospitals, 2009–2018: A quasiexperimental study. Clinical Infectious Diseases. 2020;71:1756–9. doi: 10.1093/cid/ciaa009 31922536

[39] Zaas AK, Song X, Tucker P, Perl TM. Risk factors for development of vancomycin-resistant enterococcal bloodstream infection in patients with cancer who are colonized with vancomycin-resistant enterococci. Clin Infect Dis. 2002;35:1139–46. doi: 10.1086/342904 12410472

[40] Gouliouris T, Warne B, Cartwright EJP, Bedford L, Weerasuriya CK, Raven KE, et al. Duration of exposure to multiple antibiotics is associated with increased risk of VRE bacteraemia: A nested case-control study. Journal of Antimicrobial Chemotherapy. 2018;73:1692–9. doi: 10.1093/jac/dky075 29548009 PMC5961253

[41] Kim YJ, Kim S Il, Kim YR, Lee JY, Park YJ, Kang MW. Risk factors for vancomycin-resistant enterococci infection and mortality in colonized patients on intensive care unit admission. Am J Infect Control. 2012;40:1018–9. doi: 10.1016/j.ajic.2012.01.009 22483236

