# Supplementary material for: Epidemiology and infection control of vancomycin-resistant enterococci at a German university hospital: A three-year retrospective cohort study
Source: PLoS One. 2024 Feb 26;19(2):e0297866. doi: 10.1371/journal.pone.0297866 (PMC10896503; doi:10.1371/journal.pone.0297866)
Supplement: S1 Checklist — (DOCX) [file pone.0297866.s001.docx]

STROBE Statement—checklist of items that should be included in reports of observational studies

|  | Item No. | Recommendation | Page  No. | Relevant text from manuscript |
| --- | --- | --- | --- | --- |
| **Title and abstract** | 1 | (*a*) Indicate the study’s design with a commonly used term in the title or the abstract | 1 | “…A three-year retrospective cohort study” |
|  |  | (*b*) Provide in the abstract an informative and balanced summary of what was done and what was found | 2, 3 | See Abstract |
| Introduction | | | |  |
| Background/rationale | 2 | Explain the scientific background and rationale for the investigation being reported | 4 | “…..Against this background, we attempted to better understand the local epidemiology of VRE by conducting a retrospective cohort study including all patients with VRE from 2015-2017 at Hannover Medical School (Germany), a tertiary university clinic that specializes in solid organ transplantation.” |
| Objectives | 3 | State specific objectives, including any prespecified hypotheses | 3 | “In summary, an emerging problem for hospitals and for hospital epidemiologists and infection control practitioners in Germany and worldwide is to find appropriate measures to prevent nosocomial VRE acquisition and infection” |
| Methods | | | |  |
| Study design | 4 | Present key elements of study design early in the paper | 4 | “A retrospective cohort study was conducted including all inpatients with VRE (colonization and/or infection; species: *E. faecium* and *faecalis*; January 2015 to December 2017, i.e., over 36 months).” |
| Setting | 5 | Describe the setting, locations, and relevant dates, including periods of recruitment, exposure, follow-up, and data collection | 4 | “Hannover Medical School is a university clinic in northern Germany with approximately 1,500 patient beds for the treatment of adult and pediatric patients.”  “A retrospective cohort study was conducted including all inpatients with VRE (colonization and/or infection; species: *E. faecium* and *faecalis*; January 2015 to December 2017, i.e., over 36 months).”  “VRE patients and the corresponding hospital stays (VRE cases) were identified using inhouse infection control software and the laboratory information system. A VRE patient/case either had a VRE-positive microbiologic sample during the hospital stay and/or was labeled as a known VRE carrier in the infection control software (due to VRE-positive samples in previous hospital stays or in other hospitals). Clinical and demographic data were collected from patient charts.” |
| Participants | 6 | (*a*) *Cohort study*—Give the eligibility criteria, and the sources and methods of selection of participants. Describe methods of follow-up  *Case-control study*—Give the eligibility criteria, and the sources and methods of case ascertainment and control selection. Give the rationale for the choice of cases and controls  *Cross-sectional study*—Give the eligibility criteria, and the sources and methods of selection of participants | 4 | “A retrospective cohort study was conducted including all inpatients with VRE (colonization and/or infection; species: *E. faecium* and *faecalis*; January 2015 to December 2017, i.e., over 36 months). VRE patients and the corresponding hospital stays (VRE cases) were identified using inhouse infection control software and the laboratory information system” |
|  |  | (*b*) *Cohort study*—For matched studies, give matching criteria and number of exposed and unexposed  *Case-control study*—For matched studies, give matching criteria and the number of controls per case | Not applicable | - |
| Variables | 7 | Clearly define all outcomes, exposures, predictors, potential confounders, and effect modifiers. Give diagnostic criteria, if applicable | 4, 5 | “VRE infections were classified using criteria provided by the German National Reference Center for Surveillance of Nosocomial Infections [20], which are based on the well-established CDC/NHSN definitions [21]. Briefly, the definition includes the detection of VRE in samples taken for infection diagnostics and, for some types of infection, this is combined with other parameters such as the presence of clinical signs of infection. Patients/cases not matching the infection criteria were classified as colonized. In general, a VRE acquisition (colonization/infection) on day 3 or later of the hospital stay without a known history of VRE was defined as hospital-acquired (nosocomial)”  “The incidence and incidence density of VRE were calculated as the number of VRE cases per 100 cases and per 1000 patient days, respectively. Epidemiological and clinical characteristics of cases with nosocomial VRE infection and VRE colonization were compared. Differences between the two groups were tested using the chi-square test for categorical parameters and the Wilcoxon rank sum test for continuous parameters. Numbers and percentages (categorical parameters) as well as medians, means and interquartile ranges (continuous parameters) were calculated.” |
| Data sources/ measurement | 8* | For each variable of interest, give sources of data and details of methods of assessment (measurement). Describe comparability of assessment methods if there is more than one group | 4 | “VRE patients and the corresponding hospital stays (VRE cases) were identified using inhouse infection control software and the laboratory information system. A VRE patient/case either had a VRE-positive sample during the hospital stay and/or was labeled as a known VRE carrier in the infection control software (due to VRE-positive samples in previous hospital stays or in other hospitals). Clinical and demographic data were collected from patient charts.” |
| Bias | 9 | Describe any efforts to address potential sources of bias | Not applicable |  |
| Study size | 10 | Explain how the study size was arrived at | Not applicable |  |

Continued on next page

| Quantitative variables | 11 | Explain how quantitative variables were handled in the analyses. If applicable, describe which groupings were chosen and why | 5 | “The incidence and incidence density of VRE were calculated as the number of VRE cases per 100 cases and per 1,000 patient days, respectively. Epidemiological and clinical characteristics of cases with nosocomial VRE infection and VRE colonization were compared. Differences between the two groups were tested using the chi-square test for categorical parameters and the Wilcoxon rank sum test for continuous parameters. Numbers and percentages (categorical parameters) as well as medians, means and interquartile ranges (continuous parameters) were calculated.” |
| --- | --- | --- | --- | --- |
| Statistical methods | 12 | (*a*) Describe all statistical methods, including those used to control for confounding | 5, 6 | “The incidence and incidence density of VRE were calculated as the number of VRE cases per 100 cases and per 1,000 patient days, respectively. Epidemiological and clinical characteristics of cases with nosocomial VRE infection and VRE colonization were compared. Differences between the two groups were tested using the chi-square test for categorical parameters and the Wilcoxon rank sum test for continuous parameters. Numbers and percentages (categorical parameters) as well as medians, means and interquartile ranges (continuous parameters) were calculated.  To determine independent factors (parameters) that are associated with developing a nosocomial VRE infection (compared to being/remaining VRE-colonized only), a multivariate analysis was carried out using a logistic regression model by stepwise forward variable selection. The significance level for the logistic regression was 0.05. That means, parameters with a p value <0.05 remain in the model and parameters with p value ≥0.05 were removed. For the logistic regression model, parameters that occurred during the “time at risk” were included. For being/remaining VRE-colonized, the “time at risk” was defined as either i) days from the timepoint of the first VRE colonization sample to discharge or ii) days from admission to discharge (for known VRE carriers). For nosocomial VRE infection, the “time at risk” was defined as either i) days from admission to the onset of nosocomial VRE infection (for those cases who directly developed a nosocomial VRE infection without being colonized in advance) or ii) days from the timepoint of the first VRE colonization sample to the onset of nosocomial VRE infection (for those cases who were VRE-colonized in advance of nosocomial VRE infection). To count the days, the day of admission/discharge (counted as a full day) and the day of collection of the positive VRE specimen (colonization and/or infection) were used as needed according to the above definition.  All statistical test results were considered significant at p < 0.05. The analyses were performed with SPSS 26 (IBM SPSS statistics, Somer, NY, USA) and SAS 9.4 (SAS Institute, Cary, NC, USA).” |
|  |  | (*b*) Describe any methods used to examine subgroups and interactions | See Item 12 |  |
|  |  | (*c*) Explain how missing data were addressed | Not applicable |  |
|  |  | (*d*) *Cohort study*—If applicable, explain how loss to follow-up was addressed  *Case-control study*—If applicable, explain how matching of cases and controls was addressed  *Cross-sectional study*—If applicable, describe analytical methods taking account of sampling strategy | Not applicable (retrospective study) |  |
|  |  | (*e*) Describe any sensitivity analyses | Not applicable |  |
| Results | | | | |
| Participants | 13* | (a) Report numbers of individuals at each stage of study—eg numbers potentially eligible, examined for eligibility, confirmed eligible, included in the study, completing follow-up, and analysed | 6 | “In the study period, 188,332 cases (corresponding to 137,314 individual patients; i.e., an average of 1.4 cases per patient) were recorded. These cases generated 1,381,806 patient days (mean length of stay: 7.3 days per case). There were 1,492 VRE cases corresponding to 822 individual VRE patients (1.8 cases per VRE patient).” |
|  |  | (b) Give reasons for non-participation at each stage | Not applicable |  |
|  |  | (c) Consider use of a flow diagram | Not applicable |  |
| Descriptive data | 14* | (a) Give characteristics of study participants (eg demographic, clinical, social) and information on exposures and potential confounders | 9 | See Table 1 |
|  |  | (b) Indicate number of participants with missing data for each variable of interest | Not applicable |  |
|  |  | (c) *Cohort study*—Summarise follow-up time (eg, average and total amount) | Not applicable (retrospective study) |  |
| Outcome data | 15* | *Cohort study*—Report numbers of outcome events or summary measures over time | 6,7 | See Item 13 |
|  |  | *Case-control study—*Report numbers in each exposure category, or summary measures of exposure | Not applicable |  |
|  |  | *Cross-sectional study—*Report numbers of outcome events or summary measures | Not applicable |  |
| Main results | 16 | (*a*) Give unadjusted estimates and, if applicable, confounder-adjusted estimates and their precision (eg, 95% confidence interval). Make clear which confounders were adjusted for and why they were included | 11 | See Table 2 and S1 Table |
|  |  | (*b*) Report category boundaries when continuous variables were categorized | Not applicable |  |
|  |  | (*c*) If relevant, consider translating estimates of relative risk into absolute risk for a meaningful time period | Not applicable |  |

Continued on next page

| Other analyses | 17 | Report other analyses done—eg analyses of subgroups and interactions, and sensitivity analyses | 7 | “In 912 of the 1,492 VRE cases (61.1%), there were VRE-positive samples in our hospital’s laboratory (the remaining 580 cases were known VRE carriers). The two most common positive sample sites (sample site copy strains eliminated) were rectal/stool (572 of 912, 62.7%) and urine (310 of 912, 34.0%). ….” |
| --- | --- | --- | --- | --- |
| Discussion | | | | |
| Key results | 18 | Summarise key results with reference to study objectives | 11 | “The overall occurrence of VRE in our setting (0.6% of all patients) is comparable to results from a study by Simor et al. reporting a median VRE prevalence of 0.5% in Canadian hospitals [22]. …..” |
| Limitations | 19 | Discuss limitations of the study, taking into account sources of potential bias or imprecision. Discuss both direction and magnitude of any potential bias | 15 | “Our study has strengths and limitations. Regarding limitations, the current study was conducted in Germany, which may limit its transferability to other settings, for example, due to different structural and human resource conditions. In addition, data collection was retrospective. Moreover, it was beyond the scope of our study to analyze factors associated with VRE-related mortality or to assess the economic impact of VRE. We did not use a comorbidity score to stratify the burden of existing diseases in the VRE colonization and VRE infection groups; however, length of stay and type of hospital discharge may be taken as surrogate parameters. It was not within the scope of this study to determine the molecular characteristics of the VRE isolates. Regarding strengths, we can provide a comprehensive and detailed overview of the VRE burden in a dynamic epidemic situation over a three-year period with approximately 1,500 VRE cases included. Moreover, we provide a detailed insight into the early period of the VRE increase in Northern Germany at a university clinic specialized in solid organ transplantation. Additionally, we analyzed factors independently associated with VRE infection that can be considered by healthcare workers caring for patients with a possible risk of VRE infection |
| Interpretation | 20 | Give a cautious overall interpretation of results considering objectives, limitations, multiplicity of analyses, results from similar studies, and other relevant evidence | 15 | See Item 19 and  “VRE imposed a relevant and increasing infection control burden at our tertiary care university hospital.” |
| Generalisability | 21 | Discuss the generalisability (external validity) of the study results | 15 | See Item 19 |
| Other information | |  | | |
| Funding | 22 | Give the source of funding and the role of the funders for the present study and, if applicable, for the original study on which the present article is based | Not applicable |  |

*Give information separately for cases and controls in case-control studies and, if applicable, for exposed and unexposed groups in cohort and cross-sectional studies.

**Note:** An Explanation and Elaboration article discusses each checklist item and gives methodological background and published examples of transparent reporting. The STROBE checklist is best used in conjunction with this article (freely available on the Web sites of PLoS Medicine at http://www.plosmedicine.org/, Annals of Internal Medicine at http://www.annals.org/, and Epidemiology at http://www.epidem.com/). Information on the STROBE Initiative is available at www.strobe-statement.org.
