# Supplementary material for: Epidemiology and infection control of vancomycin-resistant enterococci at a German university hospital: A three-year retrospective cohort study
Source: PLoS One. 2024 Feb 26;19(2):e0297866. doi: 10.1371/journal.pone.0297866 (PMC10896503; doi:10.1371/journal.pone.0297866)
Supplement: S1 Table — (DOCX) [file pone.0297866.s002.docx]

S1 Table. Parameters considered in the multivariable analysis.

| **Parameter** |
| --- |
| **Basic epidemiologic information** |
| Median length of “time at risk” in days |
| Median number of prior hospital stays |
| Median age in years |
| Female |
| **Distribution of cases according to specialty** |
| Anesthesia intensive care |
| Ophthalmology |
| Dermatology |
| Gastroenterology |
| Gynecology |
| Hematology & Oncology |
| Otorhinolaryngology |
| Heart and thoracic surgery |
| Infectious Diseases |
| Immunology |
| Other intensive care |
| Cardiology |
| Oral and maxillofacial surgery |
| Neurology |
| Nephrology |
| Pediatrics |
| Plastic surgery |
| Pneumology |
| Psychiatry |
| Trauma surgery |
| Urology |
| Visceral surgery |
| Dentistry |
| **Carriage of other multidrug resistant bacteria prior to VRE acquisition*** |
| Gram-negative bacteria resistant to ciprofloxacin, third generation cephalosporins and piperacillin |
| Gram-negative bacteria resistant to carbapenems |
| **Underlying diseases** |
| Heart disease |
| Lung disease |
| Liver disease |
| Gastroenterological disease |
| Oncological disease |
| Solid neoplasia |
| Dermatological disease |
| Ocular disease |
| Vessel disease |
| Kidney disease |
| Neurological disease |
| Gynecological disease |
| Urologic disease |
| Orthopedic disease |
| Thyroid disease |
| Amputation |
| Ear-Nose-Throat Disease |
| Dental disease |
| Diabetes mellitus |
| Rheumatic disease |
| Psychiatric disease |
| **Clinical characteristics** |
| Leukocytopenia (<1000/µL) |
| Hemoglobin <8g/dL |
| Central venous catheter |
| Peripheral venous catheter |
| Drainage |
| Invasive ventilation |
| Urinary catheter |
| Any surgery |
| Solid organ transplantation |
| Hematopoietic stem cell transplantation |
| Chemotherapy |

For the logistic regression model, parameters that occurred during the “time at risk” were included. For being/remaining VRE-colonized, the “time at risk” was defined as either i) days from the timepoint of the first VRE colonization sample to discharge or ii) days from admission to discharge (for known VRE carriers). For nosocomial VRE infection, the “time at risk” was defined as either i) days from admission to the onset of nosocomial VRE infection (for those cases who directly developed a nosocomial VRE infection without being colonized in advance) or ii) days from the timepoint of the first VRE colonization sample to the onset of nosocomial VRE infection (for those cases who were VRE-colonized in advance of nosocomial VRE infection).

*The parameter “MRSA” was excluded from the final model, because it could not be estimated.
